# Regulatory mechanism of LncRNAs in gonadal differentiation of hermaphroditic fish, *Monopterus* *albus*

**DOI:** 10.1186/s13293-023-00559-y

**Published:** 2023-10-25

**Authors:** Qiaomu Hu, Xueping Xia, Zitong Lian, Haifeng Tian, Zhong Li

**Affiliations:** Yangtze River Fisheries Research Institute, Chinese Academy of Fishery Sciences, Wudayuan First Road 8, Wuhan, 430223 China

**Keywords:** *Monopterusalbus*, LncRNA, DNA methylation, Sex reversal, Gene expression

## Abstract

**Background:**

*Monopterus* *albus* is a hermaphroditic fish with sex reversal from ovaries to testes via the ovotestes in the process of gonadal development, but the molecular mechanism of the sex reversal was unknown.

**Methods:**

We produced transcriptomes containing mRNAs and lncRNAs in the crucial stages of the gonad, including the ovary, ovotestis and testis. The expression of the crucial lncRNAs and their target genes was detected using qRT‒PCR and in situ hybridization. The methylation level and activity of the lncRNA promoter were analysed by applying bisulfite sequencing PCR and dual-luciferase reporter assays, respectively.

**Results:**

This effort revealed that gonadal development was a dynamic expression change. Regulatory networks of lncRNAs and their target genes were constructed through integrated analysis of lncRNA and mRNA data. The expression and DNA methylation of the lncRNAs MSTRG.38036 and MSTRG.12998 and their target genes *Psmβ8* and *Ptk2β* were detected in developing gonads and sex reversal gonads. The results showed that lncRNAs and their target genes exhibited consistent expression profiles and that the DNA methylation levels were negatively regulated lncRNA expression. Furthermore, we found that *Ptk2β* probably regulates *cyp19a1* expression via the Ptk2β/EGFR/STAT3 pathway to reprogram sex differentiation.

**Conclusions:**

This study provides novel insight from lncRNA to explore the potential molecular mechanism by which DNA methylation regulates lncRNA expression to facilitate target gene transcription to reprogram sex differentiation in *M.* *albus*, which will also enrich the sex differentiation mechanism of teleosts.

**Supplementary Information:**

The online version contains supplementary material available at 10.1186/s13293-023-00559-y.

## Highlights

- Construction of the lncRNA-mRNA interaction network in the sex differentiation.
- DNA methylation was negatively associated with lncRNA expression in developing gonads.
- Ptk2β regulate Ptk2β/EGFR/STAT3 pathway to potentially regulate sex differentiation.

**Supplementary Information:**

The online version contains supplementary material available at 10.1186/s13293-023-00559-y.

## Plain language summary

*Monopterus* *albus* is a hermaphroditic fish that undergoes sex reversal from female to male via intersex during the process of the gonadal differentiation which was an ideal model for epigenetic modification research. After laying eggs, the female *M.albus* reversal to the intersex. So that the female have a shorter stage and smaller body size which cause low egg production. In the present study, we produced the transcriptomes which contain mRNA and lncRNA in the crucial stage of the gonad including ovary, ovotestis and testis. This effort reveals that gonadal development was a dynamic expression changes. Regulatory networks of lncRNAs and its target genes were constructed though integrated analysis of lncRNA and mRNA data. We found DNA methylation was negatively associated with lncRNA (MSTRG.38036 and MSTRG.12998) expression in developing gonads. Additionally, 17α-methyltestosterone inhibit the expression of lncRNA and increase methylation. Furthermore, we found that *Ptk2β* probably regulates *cyp19a1* expression via the Ptk2β/EGFR/STAT3 pathway to reprogram sex differentiation. The present study on the gonadal differentiation of *M.* *albus* provides novel insights from lncRNA to explore potential molecular mechanism. In the future, function of the lncRNA will be further studied and the gene editing technology will be applied to cultivate the female with high fecundity to improve the yield of fish fry.

**Supplementary Information:**

The online version contains supplementary material available at 10.1186/s13293-023-00559-y.

## Introduction

Rice field eels (*Monopterus* *albus*) are a commonly known freshwater fish in China. It has a snake-like body and is widely distributed in China. Due to its high nutritive value and good palatability, wild ricefield eel has been overfished. Additionally, the wild population has also decreased sharply due to environmental damage. Altogether, the decline in natural resources, especially the shortage of offspring material, necessitates breeding. However, artificial breeding technology has succeeded and provided a useful way to increase the wild population and to improve the rice field eel industry.

Organisms of sexual reproduction have two kinds of sex: female and male. The development processes of both gonads are involved in sex determination and sex differentiation. Therefore, the mechanism of sex differentiation is one of the most fundamental biological issues. First reported in 1944, *M.* *albus* was observed to have female-to-male sex reversal [1]. In the early developmental stage, an ovary structure is observed in ricefield eel; after spawning, the ovary structure degenerates, and the male germ cells begin to develop. When ricefield eel is grown into the second developmental cycle of sexual maturity, the ovary and testis structures are observed at this stage. Finally, the structure of the ovary disappears completely, and the structure of the testis forms afterwards. To date, many differentially expressed genes have been identified and the function was studied on sex reversal in *M.* *albus* [2–7], but the mechanism involved in sex reversal is unclear.

With the development of biotechnology, many new approaches have been applied to study the mechanism of gonadal differentiation. Long noncoding RNAs (lncRNAs), as an important type of RNA, are transcripts that are more than 200 bp in length and do not encode proteins [8]. LncRNAs have lower concentrations than RNAs but have higher tissue specificity [9, 10]. Based on their relative position to protein coding genes, lncRNAs can be divided into sense, antisense, bidirectional, intronic and intergenic lncRNAs [11]. According to functional studies, lncRNAs can be divided into nuclear lncRNAs and cytoplasmic lncRNAs [12]. Accumulating evidence indicates that lncRNAs are involved in numerous biological processes, such as cell proliferation, differentiation, stem cell maintenance, RNA‒protein, RNA‒DNA and RNA‒RNA interactions [13–15]. A previous study demonstrated that lncRNA expression and function have been mainly studied in many fish involved in sex differentiation and immune response, such as Nile tilapia [16], Chinese tongue sole [17], coho salmon [18] and grass carp [19]. Recently, lncRNAs in *M.* *albus* were also identified and characterized [20]. However, the molecular mechanism of lncRNA in *M.* *albus* gonad development is still unknown.

In the present study, we produced the transcriptomes in the crucial stage of the gonad, including the ovary, ovotestis and testis. As per the expression profile and location in chromosome, two important lncRNAs and their target genes, lncRNA MSTRG.38036/Psmβ8 and MSTRG.12998/Ptk2β, were selected through integrated analysis of lncRNA and mRNA data. The lncRNAs MSTRG.38036 and MSTRG.12998 and their target genes exhibited consistent expression profiles. The promoter methylation level was negatively correlated with lncRNA expression. Dual-luciferase reporter assays suggested that *Ptk2β* regulated *cyp19a1* expression via the Ptk2β/EGFR/STAT3 pathway to reprogram sex differentiation. This study provides novel insight from long noncoding RNA (lncRNA) to explore the potential molecular mechanism by which DNA methylation regulates lncRNA expression to facilitate target gene transcription to reprogram sex differentiation.

## Materials and methods

### Animals

Healthy *M.* *albus* were collected from the Aquatic Germplasm Resources Preservation and Varieties Breeding Center of Yangtze River Fisheries Research Institute, Chinese Academy of Fishery Sciences, China. To study the molecular mechanism of gonadal differentiation, gonad from three key stages.

were collected from 3 females with the average weight of 132 g and length of 45 cm, 3 intersexes with the average weight of 121 g and length of 48 cm and 3 males with the average weight of 268 g and length of 58 g under the guidance of the Yangtze River Fisheries Research Institute Care Committee (No. 2013001). Each sample was divided into three parts: one part was frozen in liquid nitrogen and stored at − 80 °C until RNA extraction; the second was fixed in 4% paraformaldehyde (pH 7.5) for 24 h and stored in 70% ethanol to prepare for histology according to a previously described method [21]; and the third was preserved in ethanol for DNA extraction.

### Library construction and RNA-seq

Nine lncRNAs (3 biological repeats per stage) were constructed in different gonad developmental stages (female, intersex and male) in *M.* *albus.* Total RNA was extracted by applying the TRIzol method according to the manufacturer’s instructions. A NanoDrop 2000 (Thermo Fisher Scientific, USA) and Bioanalyzer 2100 (Agilent Technologies, CA) were used to detect the concentration and quality. RNA integrity was identified by agarose gel electrophoresis. Three samples of each group and equal amounts of RNA from each sample were used for RNA-seq. Ten micrograms of total RNA from each sample was treated with the Ribo-Zero™ Magnetic Kit (Epicentre, WI) to remove the rRNA, followed by reverse transcription to construct the cDNA library with the NEBNext Ultra Directional RNA Library Prep Kit (NEB, USA). Then, the prepared paired-end sequencing was performed on an Illumina Nova Seq6000 (Illumina San Diego, USA). Clean data were obtained by removing reads containing adapters, reads containing poly-N sequences and low-quality reads from the raw data. The Q20, Q30, GC content and sequence duplication level of the clean data were calculated.

### LncRNA identification

The assembled transcripts were annotated using the gff compare program. The unknown transcripts were used to screen for putative lncRNAs. The computational approaches, including CPC2/CNCI/Pfam/CPAT, were combined to classify the nonprotein coding RNA from the protein-coding RNA. The putative protein-coding RNAs were filtered out using the minimum length and exon number threshold. Transcripts with lengths greater than 200 nt and with more than two exons were selected as lncRNA candidates and further screened using CPC2/CNCI/Pfam/CPAT, which has the power to distinguish protein-coding genes from noncoding genes. In addition, different types of lncRNAs, including lincRNAs, intronic lncRNAs, antisense lncRNAs, and sense lncRNAs, were selected using cuffcompare.

### Identification of the DEGs and DELncRNA

Differential expression analysis of the two groups was performed using the DESeq R package (1.10.1). DESeq provides statistical routines for determining differential expression in digital gene expression data using a model based on the negative binomial distribution. The resulting P values were adjusted using the Benjamini–Hochberg approach for controlling the false discovery rate. Transcripts with an adjusted P value < 0.01 and absolute value of log2 (fold change) > 1 found by DESeq were considered differentially expressed.

### Gene function analysis

Gene Ontology (GO) enrichment analysis of the differentially expressed transcripts was implemented by the topGO R package based on Wallenius noncentral hypergeometric distribution. KOBAS software was used to test the statistical enrichment of differentially expressed transcripts in KEGG pathways [22]. *P* < 0.05 represents a significant difference.

### Construction of the lncRNA‒mRNA interaction network

Based on the interaction mode between lncRNAs and their target genes, we applied two prediction methods. (1) LncRNAs regulate the expression of adjacent genes. The adjacent genes within 100 kb of lncRNAs are predicted to be their target genes mainly based on the positional relationship between lncRNAs and genes. (2) The target genes of lncRNAs were predicted by analysing the correlation between the expression of lncRNAs and mRNAs among samples. The genomic structure and chromosomal location of the candidate lncRNA and its target gene were analysed as per the reported genome data [23] using Integrative Genomics Viewer software.

### Quantitative real-time PCR

Total RNA was extracted from the ovary, testis and ovotestis using the TRIzol method. The extracted RNA without genomic DNA was transcribed into cDNA with random hexamers for mRNA and lncRNA. qRT‒PCR was performed on an ABI Q5 real-time PCR system (Applied Biosystems, USA) using SYBR Premix Ex Taq (Takara, China) with beta-actin as an internal control as previously described [24]. All qRT‒PCR assays were performed on three samples, and each sample was repeated three times to obtain the cycle threshold. Finally, the expression of all mRNAs and lncRNAs were calculated using the 2^−ΔΔCT^ method.

### In situ hybridization

To assess the expression of MSTRG.12998 and MSTRG.38036 transcripts and their target genes in gonadal cells, primers were designed according to the sequences to amplify the T7 promoter sequence with a synthetic probe (Additional file 1: Table S1). PCR products were purified by a QIAquick Gel Extraction Kit (QIAGEN, Germany). The MEGAshortscript T7 High Yield Transcription Kit (ThermoFisher Scientific, USA) and DIG RNA labelling mix (Roche, Switzerland) were used to obtain probes. According to a previous description [25], anti-digoxigenin-AP Fab fragments (Roche, Switzerland) were used as the antibody, and BCIP/NBT (Beyotime, China) was used to detect the positive signal.

### 17α-Methyltestosterone treatment

17α-methyltestosterone (MT) was used to treat *M.* *albus* at 60 dpf to produce sex reversal in *M.* *albus* as described in previous studies[26], and no treatment was applied to the control group. Larvae were immersed in water containing MT at concentrations of 100 µg/L (MT1), 200 µg/L (MT2) and 300 µg/L (MT3) for approximately 12 h daily. Additionally, the larvae were fed using daphnia at a concentration of 200 µg/g MT. The gonads were collected after treatment for two months and divided into three parts. One part was preserved in 4% paraformaldehyde (pH 7.5) to prepare tissue sections, the second part was frozen in liquid nitrogen and then stored at – 80 °C for RNA extraction, and the last part was stored in ethanol for DNA extraction.

### Treatment with ZD6474

Forty healthy *M.* *albus* individuals weighing 20 g were collected for treatment with ZD6474 which was a tyrosine kinase inhibitor. ZD6474 (10 mg, MCE, USA) was dissolved in 1 ml DMSO to prepare a solution of 10 mg/ml according to the manufacturer's instructions. Then, according to the manufacturer's instructions, the injection solution was prepared with 1 ml of 10 mg/ml ZD6474, 4 ml PEG300, 0.5 ml Tween 80 and 4.5 ml physiological saline to a final concentration of 1 mg/ml ZD6474. Each individual was injected with 300 µl of 1 mg/ml ZD6474, and the second group was injected with an equal amount of solution without ZD6474 as the control. After injection for 24, 48, 72, 96 h, gonads were collected from at least three individuals, frozen in liquid nitrogen and then stored at − 80 °C for RNA extraction.

### Bisulfite PCR methylation analysis

Genomic DNA was extracted from ovary, ovotestis and testis tissues, and at least 15 individuals were used in each group using the TIANamp Genomic DNA Kit (Tiangen, China). Concentration and integrity were identified by an Agilent 2100 Bioanalyzer (Agilent, USA) and agarose gel electrophoresis, respectively. Equal amounts of DNA were mixed in the same group and treated using a DNA methylation kit (Zymo, USA) following the manufacturer's protocol. PCR amplification was conducted using treated DNA as the template, and primers were designed by online MethPrimer design software (http://www.urogene.org/methprimer/). The PCR products were purified and cloned into the PMD-18 T vector. A total of 15–20 positive clones from each group were sequenced, and the methylation level was analysed using the DNA methylation analysis platform (http://services.ibc.uni-stuttgart.de/BDPC/BISMA/).

### Plasmid constructs

The double-restriction endonucleases NheI and XhoI (NEB, USA) were used to construct the expression plasmid pcDNA3.1-STAT3. The promoter sequence of *cyp19a1* was found in the genomic database. Three deletion fragments (1599 bp, 1097 bp and 447 bp) of the promoter were amplified from genomic DNA according to the designed primers (Additional file 1: Table S1), and the PCR products were purified and cloned into the pGL3-basic vector (Promega, USA) using KpnI and HindIII. Site-directed mutagenesis for the stat3 binding sites was performed using a Fast Site-Directed Mutagenesis Kit (Tiangen, China), and the primers are described in Additional file 1: Table S1.

### Dual-luciferase reporter assays

HEK293T cells were obtained from the Center of Animal Science and Animal Medicine, Shandong Agricultural University. The cells were cultured at 37 °C in DMEM (Thermo Fisher Scientific, USA) containing 10% FBS (BioInd, Israel) and 1% P/S (Thermo Fisher Scientific, USA). Before transfection, the cells were seeded onto 24-well plates. When the concentration reached 1 × 10^5^ per well, the DMEM was removed, and Opti-MEM was added to incubate the cells. Then, 500 ng recombinant constructs and 50 ng pRL-TK were cotransfected into the cells in 400 µl opti-MEM medium using Lipofectamine TM 3000 (Invitrogen, USA) according the manufacturer’s instructions and incubated at 37 °C for 6 h. After that, opti-MEM medium was removed, and DMEM was incubated for 48 h. Cells were collected, and luciferase activity assays were performed using a dual-luciferase kit (Promega, USA) on a Flexstation 3.

### Statistics

All the statistical tests were performed by using the SPSS 22.0 (IBM). The expression and methylation data of developing gonad were analysed by one-way ANOVA followed by Duncan’s multiple comparison tests. Independent sample t test was used to detect the difference of expression between control and experiment group treated by MT or ZD6474, differences in the mean methylation level between control and MT treated group and differences in the luciferase activity. Differences in the ratio of methylated to unmethylated CpG at each site were assessed by a chi-square test followed by Fisher's exact test. A P value of less than 0.05 was considered significant.

## Results

### RNA-seq in *M. albus*

To explore the putative candidate lncRNAs related to sex reversal, 18 RNA-seq libraries were constructed (nine for lncRNAs and mRNAs). A total of 350,071,576, 339,654,594 and 330,891,984 clean reads were obtained from 52.24G, 50.58G and 49.42G clean data in ovotestis, ovary and testis tissues (Additional file 1: Fig S1), respectively (Table 1). The data were deposited in the National Genomics Data Center under the accession number CRA007120. All clean reads were assembled and mapped to the reference genome of *M.* *albus* [23]. A range of 82.08–92.34% reads were mapped to the genome among the 9 samples (Table 1). A total of 46,994,829, 33,212,365 and 37,683,079 clean reads were generated from ovotestis, ovary and testis, respectively, and the data were deposited in the National Genomics Data Center under the accession number CRA007125.

**Table 1:** Summary of LncRNA-seq dataset

Sample ID	Clean reads (*n*)	Clean base (*G*)	Mapped reads	Mapped rate (%)	Q20 (%)	Q30 (%)
OVT36	114,076,308	17.03	95,483,267	83.70%	97.54	93.56
OVT46	121,430,968	18.12	106,896,037	88.03%	97.69	93.84
OVT48	114,564,300	17.09	96,394,766	84.14%	97.54	93.56
OV3	110,642,888	16.56	102,070,868	92.25%	98.48	95.71
OV4	115,562,548	17.29	106,707,764	92.34%	98.58	95.91
OV6	113,449,158	16.98	100,571,879	88.65%	98.29	95.41
TE5	110,378,442	16.47	92,301,970	83.62%	98.27	95.30
TE6	108,000,138	16.13	88,650,132	82.08%	98.11	94.94
TE7	112,513,404	16.82	92,960,416	82.62%	98.21	95.18

### LncRNA and mRNA identification

**Fig. 1:**
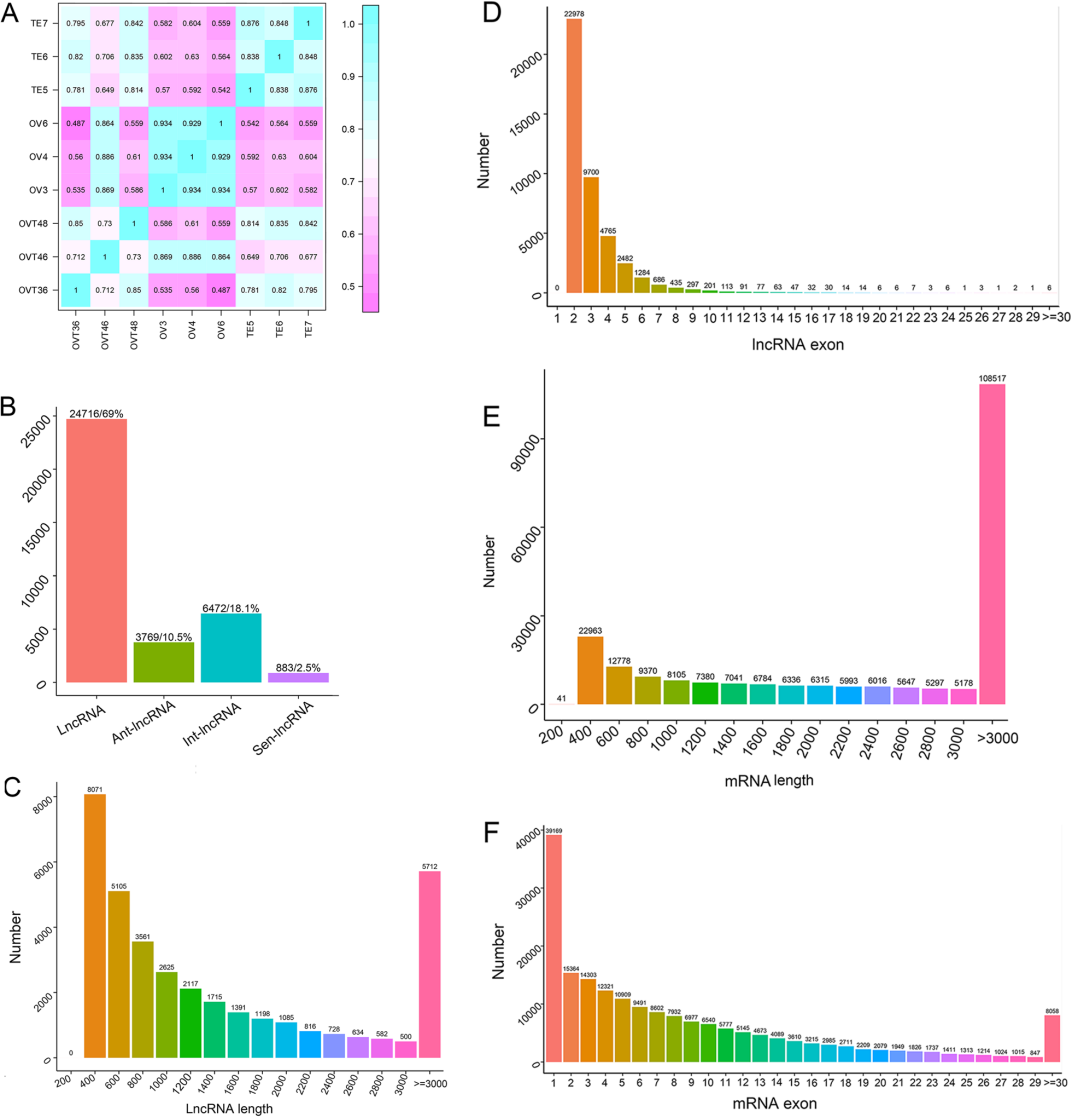
Identification of mRNA and lncRNA in the developing gonad of *Monopterus* *albus.* **A** Pearson correlation coefficient among ovary (OV), ovotestis (OVT) and testis (TE); **B** number of each kind of lncRNA; **C** length distribution of lncRNA; **D** exon number distribution of lncRNA; **E** length distribution of mRNA; **F** exon number distribution of mRNA

To assess the similarity of the gonad samples, pairwise Pearson’s correlation coefficients were calculated between every two samples. The correlation coefficient was > 0.929 in the ovary group, > 0.838 in the testis group and > 0.712 in the ovotestis group (Fig. 1A). Four methods, CPC, CNCI, CPAT, and PFAM, were used to identify lncRNAs, and 25840 overlapping lncRNAs were obtained, including 24716 lncRNAs, 3769 antisense lncRNAs, 6472 intronic lncRNAs and 883 sense lncRNAs (Fig. 1B). The length of the lncRNA was calculated and showed that most of the lncRNAs were assembled at 400 bp. With increasing length, the number of lncRNAs decreased (Fig. 1C). The number of lncRNA exons was analysed, and the results showed that 97% of the lncRNAs had fewer than 5 exons (Fig. 1D). The mRNA sequence results showed that small amount of the mRNA lengths were more than 3000 bp (Fig. 1E). mRNAs with more than three exons exceeded 80% (Fig. 1F).

### Identification of DEGs and DELncRNA

**Fig. 2:**
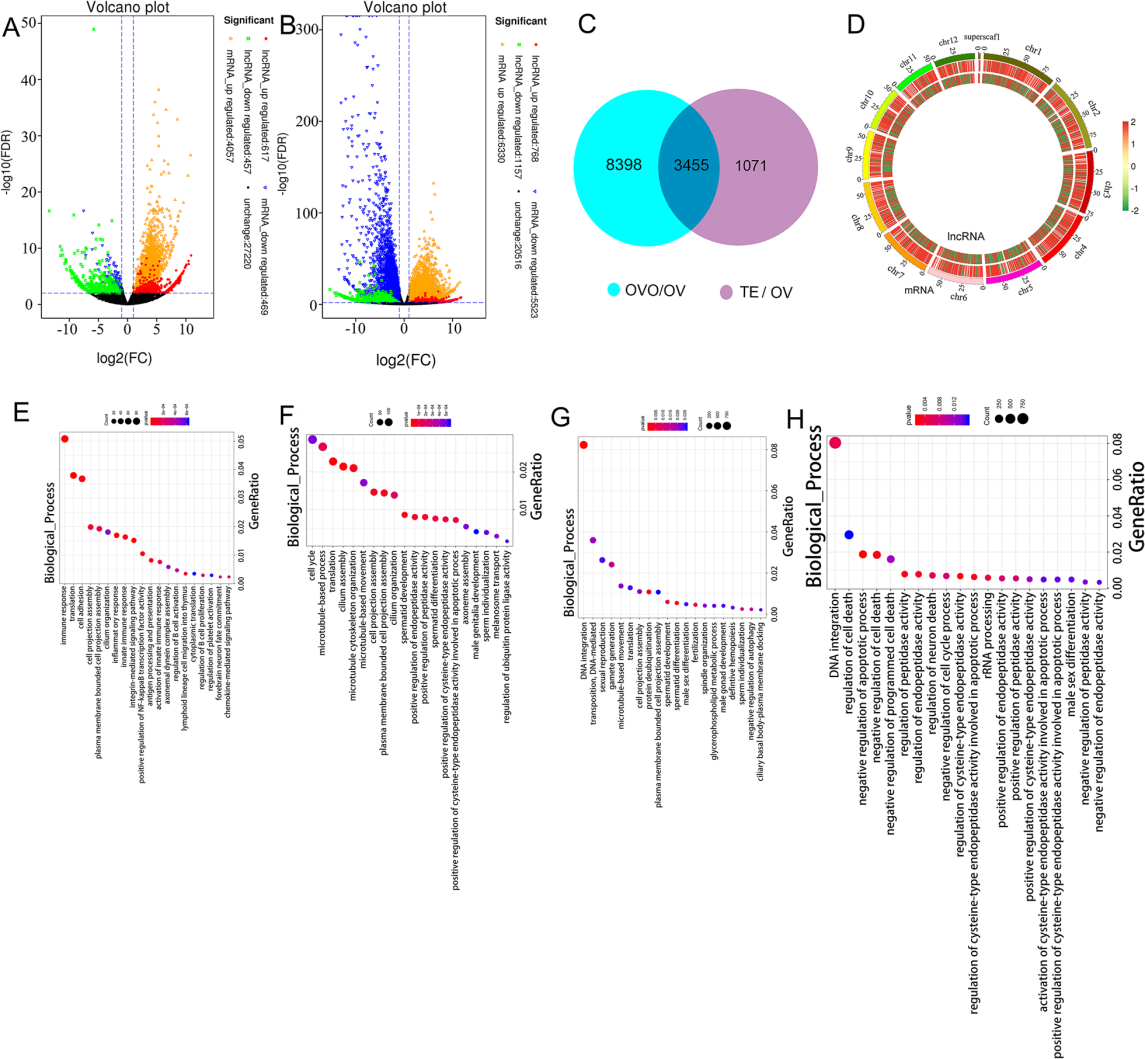
Identification of DEGs and DELncRNAs in the developing gonad of *Monopterus* *albus.* Volcanos plot for the DEGs and DELncRNAs between OVT vs OV (**A**), TE vs OV (**B**). **C** Venn diagram for the overlap genes between OVT vs OV, and TE vs OV. **D** location of the DEGs and DELncRNAs on the genome. GO enrichment for the DEGs between OVT vs OV (**E**), TE vs OV (**F**), GO enrichment for the DELncRNAs between OVT vs OV (**G**), TE vs OV (**H**). The expression level differing at least twofold in the gonad between the two groups was considered as the DEGs or DELncRNAs

To detect the expression difference among the ovary, ovotestis and testis groups, DEGs and DELncRNAs were analysed between the OVT/OV and TE/OV groups. In the OVT/OV group, 4562 DEGs were detected, including 4057 upregulated genes and 469 downregulated genes, with 1074 DElncRNAs, including 617 upregulated lncRNAs and 457 downregulated lncRNAs (Fig. 2A). In the TE/OV group, 11853 DEGs were detected, including 6330 upregulated genes and 5523 downregulated genes, with 1925 DELncRNAs, including 768 upregulated lncRNAs and 1157 downregulated lncRNAs (Fig. 2B). A total of 3455 overlapping genes were detected between the two groups (Fig. 2C). To locate the differentially expressed mRNAs and lncRNAs in the genome, the distribution of the DEGs and DELncRNAs was calculated across the genome in the OVT/OV and TE/OV groups (Fig. 2D). To better understand the mechanism of the regulatory network, GO enrichment analysis was performed for the DEGs and DELncRNAs The DEGs in the OVT/OV groups were categorized as immune response, translation and cell adhesion (Fig. 2E), and the DEGs in the TE/OV groups were mainly classified as cell cycle, translation and cilium assembly (Fig. 2F). The DELncRNA in the OVT/OV groups was mainly classified as positive regulation of cell death (Fig. 2G), and in the TE/OV groups DELncRNA was mainly distributed in terms of cellular component morphogenesis and regulation of response to stress (Fig. 2H).

### Construction of the lncRNA‒mRNA interaction network

According to the location and co-expression profile of the lncRNA and mRNA, 354 lncRNAs and their target genes were located on the same chromosome as the co-expression profile (Table S2). Due to the expression profile of target genes, 15 pairs of lncRNAs and its target genes showed potential function in sex reversal during the gonadal development (Fig. S1). Out of the 15 pairs of lncRNAs and its target genes, 4 pairs (MSTRG.38036/Psmβ8, MSTRG.12998/Ptk2β, MSTRG.24970/newGene_11061, and MSTRG.34402/newGene_17932) had consistent expression patterns between lncRNAs and their target genes (Additional file 2: Fig S2), with the highest expression in OVTs and low expression in OVs and TEs. According to the annotation of target genes, MSTRG.38036/Psmβ8 and MSTRG.12998/Ptk2β were selected for further study.

### Identification and characterization of the lncRNAs

The 1808 bp and 551 bp full-length sequences of MSTRG.38036 and MSTRG.12998 transcripts were screened from the genomic and transcriptomic data (Additional file 3: Fig. S3A, C). MSTRG.38036 and its target gene proteasome subunit beta type-8 (Psmβ8) were located on chromosome 3, and MSTRG.38036 contained 3 exons (Additional file 3: Fig. S3B). Additionally, MSTRG.12998 and its target gene protein tyrosine kinase 2β (Ptk2β) were located on chromosome 10, and MSTRG.12998 contained 2 exons (Additional file 3: Fig. S3D).

### DNA methylation was negatively associated with MSTRG.38036 and MSTRG.12998 expression in developing gonads

**Fig. 3:**
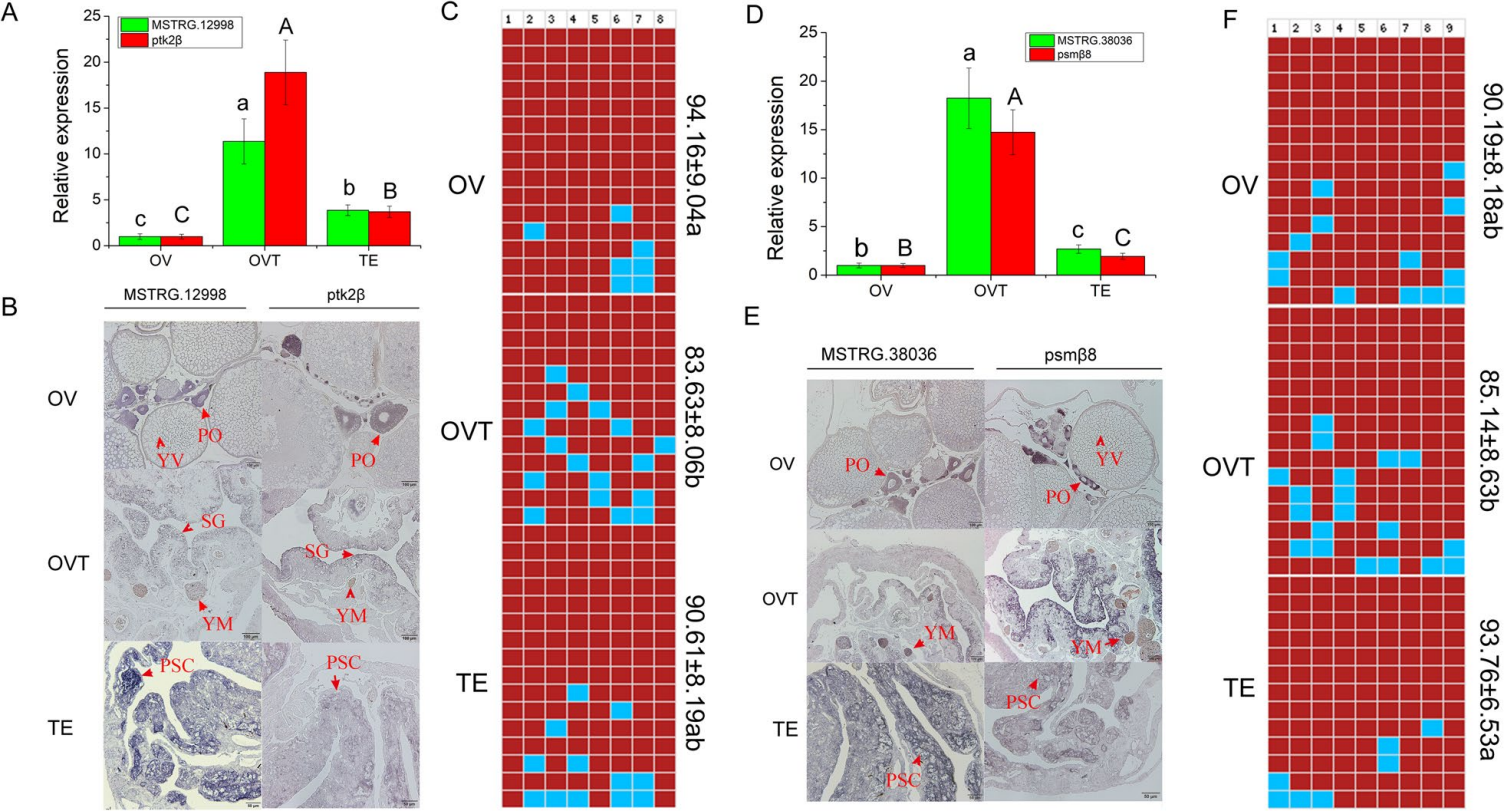
Expression and methylation level of LncRNAs and there target genes in ovary, ovariotestis and testis of *Monopterus* *albus*. **A** expression profile of MSTRG.12998 and ptk2β in ovary, ovariotestis and testis detected by qRT-PCR; **B** expression location of MSTRG.12998 and ptk2β in ovary, ovariotestis and testis detected by In Situ Hybridization; **C** DNA methylation level of MSTRG.12998 in ovary, ovariotestis and testis of *Monopterus* *albus*. **D** expression profile of MSTRG.38036 and psmβ8 in ovary, ovariotestis and testis detected by qRT-PCR; **E** expression location of MSTRG.38036 and psmβ8 in ovary, ovariotestis and testis detected by In Situ Hybridization; **F** DNA methylation level of MSTRG.38036 and psmβ8 in ovary, ovariotestis and testis of *Monopterus* *albus*. The different lowercase letters indicated the significant difference in lncRNAs expression between gonad of different stage (*P* < 0.05). The different capital letters indicated the significant difference in target genes expression between gonad of different stage (*P* < 0.05). In Fig. 4C and F, top row indicated the lncRNA promoter methylation sites and the numerical values in the right label indicated the mean methylation level of each group. *PO* primary oocyte, *PSG* primary spermatocyte, *YM* Yolk mass, *YV* Yolk vesicle, *SG* spermatogonium

To detect the regulatory mechanism of MSTRG.12998 and MSTRG.38036 in gonadal development, we analysed the association between RNA transcription and DNA methylation. We found a region around the promoter of MSTRG.38036 and MSTRG.12998 with high CpG. The methylation primers were designed at both ends of the methylation island according to the promoter sequences (Additional file 6: Table S1). We found that the MSTRG.12998 and MSTRG.38036 expression profiles were the same as those of their target genes *Ptk2β* and *Psmβ8*, with the highest expression in the ovotestis and low expression in the ovary and testis (Fig. 3A, D). To detect the distribution of MSTRG.12998, MSTRG.38036, and their target genes in gonads, in situ hybridization was used to identify their expression in ovary, ovotestis and testis cells. A sense probe was used as the negative control, and no signal was found (Additional file 4: Fig. S4). We found that the distribution of MSTRG.12998 and its target gene *Ptk2β* was similar in the gonads (Fig. 3B). Additionally, MSTRG.38036 and its target gene *Psmβ8* shared a consistent expression profile in gonad development (Fig. 3D). The distributions of MSTRG.38036 and Psmβ8 in gonads were also detected, and they were similar to that of MSTRG.12998 and its target gene (Fig. 3E). The methylation levels of the MSTRG.12998 and MSTRG.38036 promoters were detected in the same sample during gonad development. We found that the lowest methylation levels of the MSTRG.12998 (Additional file 6: Table S3) and MSTRG.38036 (Additional file 6: Table S4) promoters were exhibited in OVT (Fig. 3C, F), while significantly high methylation levels were observed in OV and TE in the MSTRG.12998 and MSTRG.38036 promoters, respectively (Fig. 3C, F). From the results, we found that methylation status was negatively associated with gene expression.

### 17α-Methyltestosterone increase the expression of lncRNA and decrease methylation

**Fig. 4:**
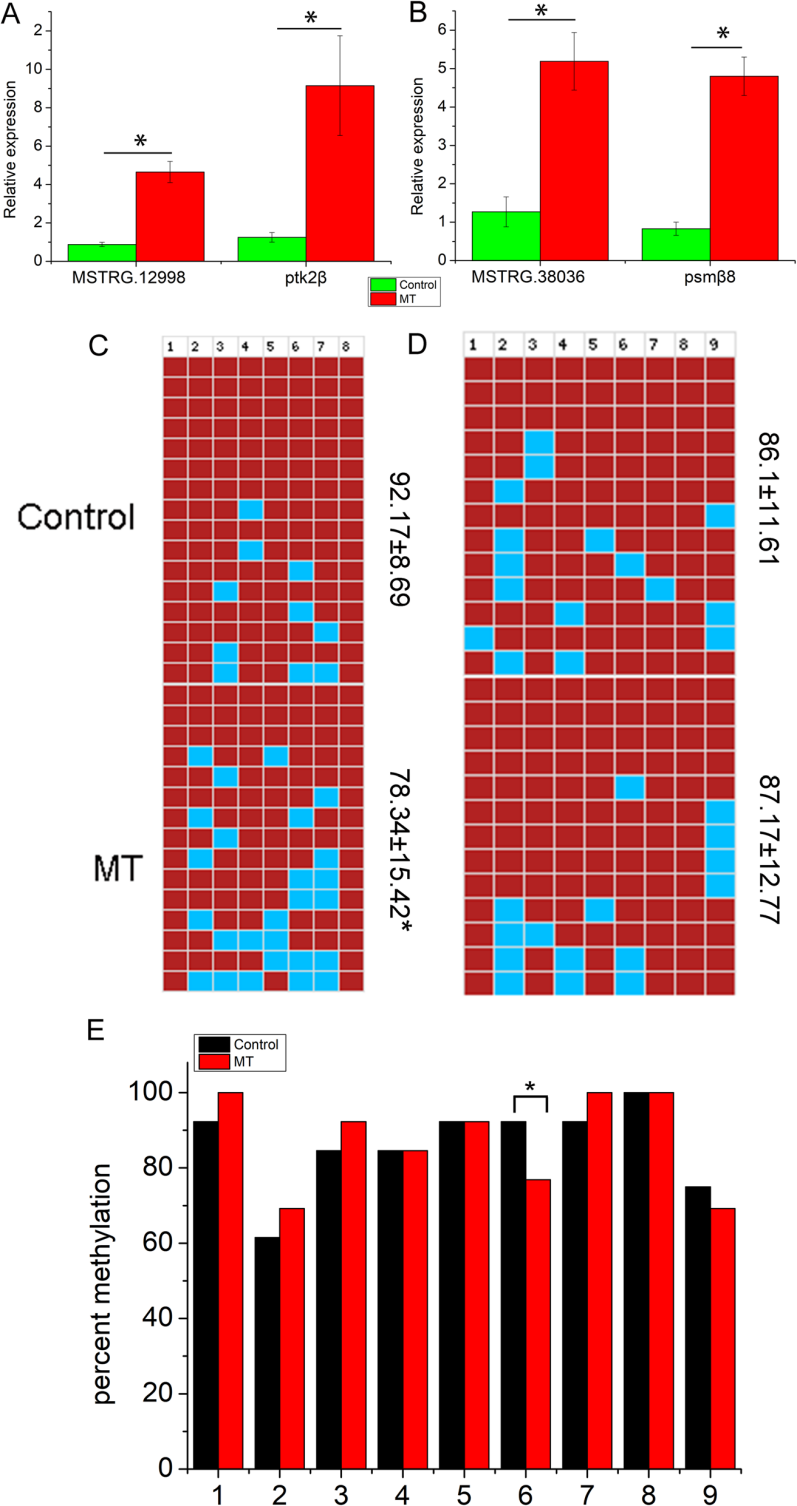
Expression and methylation level of LncRNAs in ovary and degenerated ovary treated by MT. Expression profile of MSTRG.12998/ptk2β (**A**) and MSTRG.38036/psmβ8 (**B**) in ovary and degenerated ovary; Mathylation level of MSTRG.12998 (**C**) and MSTRG.38036 (**D**) promoter in ovary and degenerated ovary; **E** mathylation level of each CpG site in MSTRG.38036 promoter in ovary and degenerated ovary. *P* < 0.05 indicate significantly difference, which was marked by *. In Fig. 5C and D, top row indicated the lncRNA promoter methylation sites and the numerical values in the right label indicated the mean methylation level of each group

To further detect the relationship of methylation level and gene expression in gonadal development, the DNA methylation level and expression profile of MSTRG.12998 and MSTRG.38036 were detected after MT treatment. The expression of MSTRG.12998, MSTRG.38036 and their target genes *Ptk2β* and *Psmβ8* was upregulated after MT treatment (Fig. 4A, B). The methylation status of the MSTRG.12998 and MSTRG.38036 promoters showed that the methylation level of the MSTRG.12998 promoter was significantly decreased (Additional file 6: Table S5, Fig. 4C , P <  0.05), while there was no significant difference in the MSTRG.38036 promoter after MT treatment (Additional file 6: Table S6, Fig. 4D, P>  0.05). However, we found that the methylation level of CpG site 6 in the MSTRG.38036 promoter was significantly decreased (Fig. 4E, P <0.05). Methylation status negatively regulates gene expression in gonadal development.

### Ptk2β regulate Ptk2β/EGFR/STAT3 pathway to potentially regulate sex differentiation

**Fig. 5:**
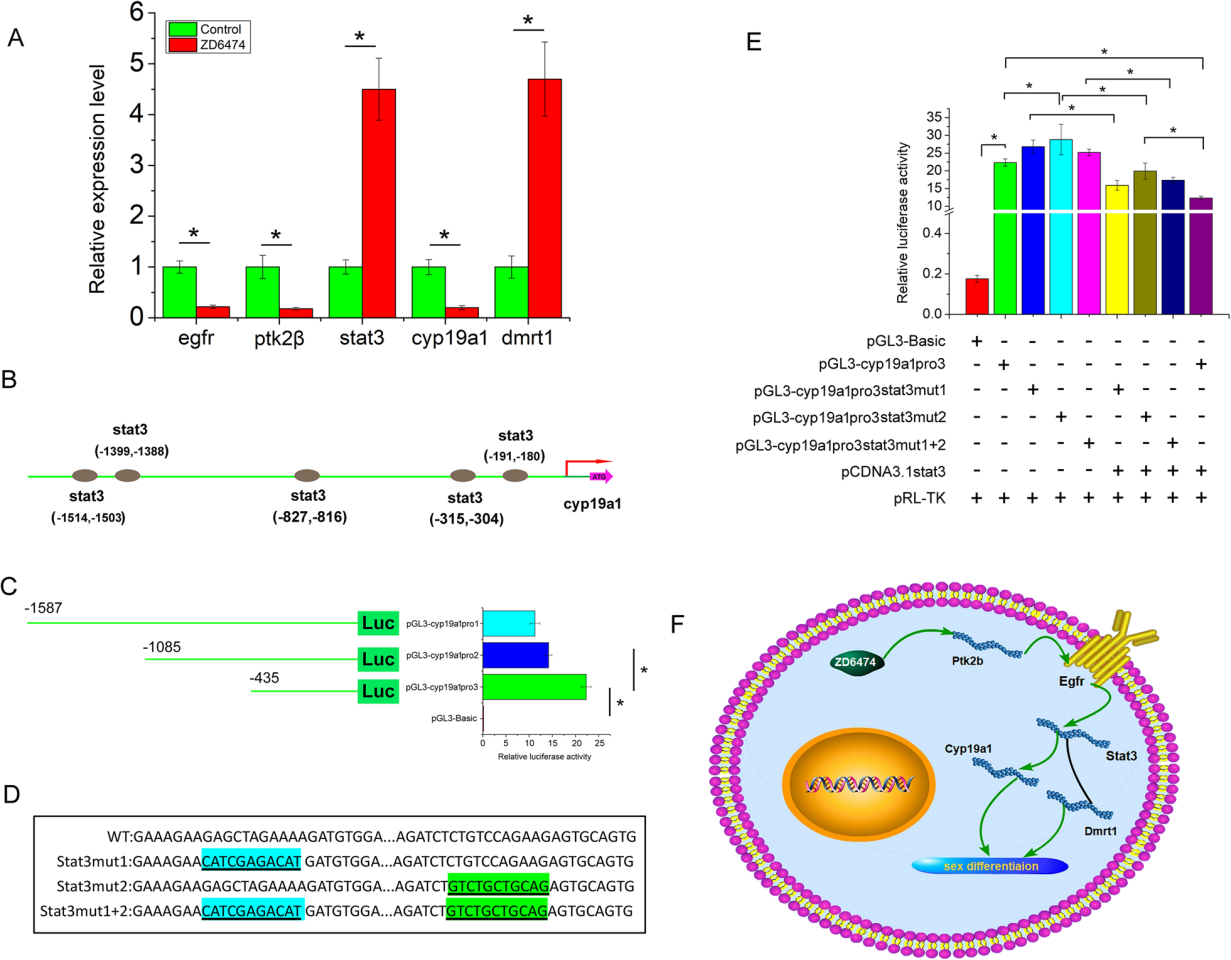
Deduced regulation mechanism of ptk2β in sex differentiation by Ptk2β/EGFR/STAT3 pathway. **A** Expression profile of the sex related gene after ZD6474 treatment in vivo; **B** Schematic showing the stat3 binding sites in cyp19a1 promoter; **C** Luciferase assay showing the activity of deletions constructs; **D** Schematic showing the mutation of stat3 and the wild type; **E** Luciferase assay showing site mutation of promoter in 293 T cells; **F** Diagram illustrating the hypothetical mechanism of ptk2β during sex differentiation in *M.* *albus* by Ptk2β/EGFR/STAT3 pathway. The mean ± SEM was from three independent experiments, *P < 0.05 show significantly difference

To detect the role of *Ptk2β* in gonadal development, ZD6474, an inhibitor of protein tyrosine kinase, was used to inhibit the expression of *Ptk2β*. Expression of Ptk2β were detected after treatment of 24, 48, 72, 96 h and the best treatment effect was observed at 72 h with the lowest expression of Ptk2β (Additional file 5: Fig S5). *cyp19a1* was a key gene to regulate the level of estrogen in sex differentiation and the promoter of *cyp19a1* was analyzed. Signal transducer and activator of transcription 3 (*stat3*) was found to be an important transcription factor, and many binding sites were predicted in the *cyp19a1* promoter using JASPAR online software. As per a previous report, the Ptk2β/EGFR/STAT3 pathway is involved in many physiological processes. Does the pathway play a role in gonadal development? After ZD6474 treatment, the expression of *Ptk2β*, *egfr* (XM_026312839) and *cyp19a1* (EU841366) was significantly decreased (Fig. 5A, P < 0.05), while the expression of *stat3* (XM_020607298.1) and *dmrt1a* (AF421347) was significantly increased (Fig. 5A, P < 0.05). To determine the exact binding sites of *stat3* in the *cyp19a1* promoter, a luciferase reporter assay with a series of deletions was conducted, and the luciferase activities were significantly higher than those of the basic group in the three deletion constructs (Fig. 5C, P < 0.05). The luciferase activities showed that key regulatory elements ranged from -1 to -435 and contained two stat3 binding sites. To further determine the regulatory role of *stat3* in *cyp19a1* expression, site mutants were constructed using pGL3-cyp19a1pro3 as the template. Three mutants, stat3mut1, stat3mut2 and stat3mut1 + 2, were obtained (Fig. 5D). Luciferase activities were significantly decreased after *stat3* binding to pGL3-cyp19a1pro3 (Fig. 5E, P < 0.05). When one or two binding sites were mutated, the luciferase activities exhibited no significant differences among the groups (Fig. 5E, P >  0.05). However, after *stat3* binding to the mutation of pGL3-cyp19a1pro3, we found that the luciferase activities were significantly increased in the pGL3-cyp19a1pro3stat3mut2/pGL3-cyp19a1pro3 group (Fig. 5E, P < 0.05), while no significant difference was observed in the pGL3-cyp19a1pro3stat3mut1/pGL3-cyp19a1pro3 and pGL3-cyp19a1pro3stat3mut1 + 2/pGL3-cyp19a1pro3 groups (Fig. 5E, P > 0.05).

These data suggest that *stat3* binding site 2 played a role in *stat3* regulation, while binding site 1 did not. Taken together, we speculate that ZD6474 inhibited *Ptk2β* expression to affect the Ptk2β/EGFR/STAT3 pathway to regulate *cyp19a1* expression by *stat3* binding site 2 in the process of gonadal development (Fig. 5F).

## Discussion

In the present study, *M.* *albus,* *a* classic sex reversal fish*,* was used as a good model species to perform epigenetic modification, especially for lncRNAs, in the process of gonadal development. We produced a transcriptome including mRNA and lncRNA of the key gonad stages in the ovary, ovotestis and testis. The expression profiles of mRNAs and lncRNAs were compared, and lncRNAs and their target genes were screened according to the location and co-expression profile. After a series of evaluation tests, we found that MSTRG.38036/Psmβ8 and MSTRG.12998/Ptk2β exhibited a co-expression profile, and their expression was significantly upregulated from the ovary to the ovotestis, while their expression decreased from the ovotestis to the testis, suggesting that the expression profile changed during the gonadal development process. According to the expression characteristics of sex differentiation genes from a previous report [27, 28], we propose a hypothesis where they both involved sex reversal during gonadal development.

To verify this hypothesis, the expression patterns of MSTRG.38036/Psmβ8 and MSTRG.12998/Ptk2β were compared, and the methylation status of MSTRG.38036 and MSTRG.12998 was detected during gonadal development. Furthermore, the expression patterns and methylation status were also detected during sex reversal after *M.* *albus* larvae were treated with MT. Moreover, a dual-luciferase reporter assay revealed that *Ptk2β* regulates *cyp19a1* expression through the Ptk2β/EGFR/STAT3 pathways to be involved in sex differentiation.

Noncoding RNAs, once considered to be “transcriptional noise”, were recently shown to have biological functions. The function of lncRNAs has been reported to regulate development and disease in biological processes [29–32]. Previous reports have demonstrated that lncRNAs have important roles in gonadal development [33–35]. In *M.* *albus,* noncoding RNAs have been identified and characterized [20, 36]*.* Until now, the lncRNA regulatory mechanism in gonadal development has remained unclear. In the present study, we produced a transcriptome in the key stage to screen critical lncRNAs and investigate the regulatory mechanism of candidate lncRNAs in gonadal development. In mice, lncRNA *Xist* binds to a critical site in the *Xist* gene body and silences a series of genes from this site to the rest of the X chromosome to inactivate the X chromosome [37–39]. Many researchers have expected the functions of lncRNAs in gonadal development and reproduction to be conserved. Conversely, most lncRNAs are differentially expressed in mammalian gonads, and only a small number have a specific role in gonadal development [40–45], such as testis-specific lncRNA regulate steroidogenesis [45]. In mice, after knockout of the lncRNA Tslrn1, the sperm count was reduced to 20%, but there was no change in fertility [46]. Until now, in mice, to our knowledge, only lncRNA Tug1 has shown significant male fertility roles, while lncRNA Tug1 is replaced with LacZ, which could lead to morphological defects in sperm and complete sterility [46]. Unlike mammals, the regulatory mechanism of lncRNAs has been reported very rarely in fish. Recently, in *Cynoglossus* *semilaevis*, Tang et al. found that the miRNA cse-miR-196 binds to circdmrt1 and the lncRNA AMSDT to upregulate the expression of the *gsdf* gene to facilitate testis differentiation [47]. In the present study, 15 pairs of candidate lncRNAs and their target genes were identified, and lncRNA MSTRG.38036 and MSTRG.12998, as per the expression profile and its target gene function, were selected for further study. The lncRNAs MSTRG.38036 and MSTRG.12998 and their target genes share a consistent expression profile, with the highest expression in the ovotestis and low expression in the ovary and testis. After spawning by female *M.* *albus*, the ovary degenerates, the testis begins to develop, and the *M.* *albus* enters the intersexual stage. From a previous report, the male sex-determining gene exhibited significantly high expression when the testis began to develop [48, 49]. Thus, the expression profile suggested that lncRNA MSTRG.38036, MSTRG.12998 and its target genes were potentially involved in sex reversal. For further verification, MT was used to treat the larvae of *M.* *albus.* We found that the ovary was degenerated after MT treatment for two months. However, in another report, we observed that *M.* *albus* larvae treated with an aromatase inhibitor for four months led to ovary reversal into testis [50]. The results indicate that an aromatase inhibitor could cause sex reversal in *M.* *albus,* but adequate treatment time is needed. The expression of the lncRNAs MSTRG.38036 and MSTRG.12998 and their target genes was significantly upregulated after MT treatment (Fig. 4). The DNA methylation levels of the lncRNA MSTRG.38036 and MSTRG.12998 promoters were assessed in developing gonads and sex reversal ovaries. The methylation status was dynamic, and the methylation level was negatively associated with gene expression. These results suggested that DNA methylation probably inhibited lncRNA MSTRG.38036 and MSTRG.12998 expression. Numerous previous reports have shown that DNA methylation regulates gene expression to reprogram sex [51, 52]. For further study, the expression of *Ptk2β* was repressed using ZD6474, which is an inhibitor of protein tyrosine kinases [53]. After ZD6474 treatment, the expression of genes involved in the Ptk2β/EGFR/STAT3 pathways was significantly changed (Fig. 5A). *Ptk2β* is a protein tyrosine kinase, and epidermal growth factor receptor (EGFR) functions as the receptor of *Ptk2β* [54]. When the expression of *Ptk2β* was downregulated, EGFR expression was also downregulated. Thus, the expression of genes involved in the Ptk2β/EGFR/STAT3 pathway was changed. Signal transducer and activator of transcription 3(*stat3*) binding sites were found in the promoter region of *cyp19a1*. Does *stat3* regulate *cyp19a1* expression? To verify this hypothesis, dual-luciferase reporter assays were conducted, and we found that *stat3* binds to the promoter region of *cyp19a1* and inhibits *cyp19a1* expression. Thus, we speculate that *Ptk2β* regulates *cyp19a1* expression to reprogram sex differentiation. Taken together, we identified a potential regulatory pathway of *Ptk2β* in sex differentiation.

## Conclusions

We produced transcriptomes containing mRNAs and lncRNAs in the crucial stages of the gonads, including the ovary, ovotestis and testis. This effort revealed that gonadal development is a dynamic expression change. Regulatory networks of lncRNAs and their target genes were constructed through an integrated analysis of lncRNA and mRNA data. The expression and DNA methylation of the lncRNAs MSTRG.38036 and MSTRG.12998 and their target genes *Psmβ8* and *Ptk2β* were detected in developing gonads and sex reversal gonads. The results showed that lncRNAs and their target genes exhibited consistent expression profiles and that the DNA methylation levels were negatively correlated with lncRNA expression. Furthermore, the dual-luciferase reporter assays showed that *Ptk2β* probably regulates *cyp19a1* expression via the Ptk2β/EGFR/STAT3 pathway to reprogram sex differentiation. This study provides novel insight from lncRNA to explore the potential molecular mechanism by which DNA methylation regulates lncRNA expression to facilitate target gene transcription to reprogram sex differentiation in *M.* *albus*, which will also elucidate the sex differentiation mechanism of teleosts.

### Perspectives and significance

*Monopterus* *albus* is a hermaphroditic fish that undergoes sex reversal from female to male via intersex during the process of the gonadal differentiation which was an ideal model for epigenetic modification research. The present study on the gonadal differentiation of *M.albus* provides novel insights from lncRNA to explore potential molecular mechanism that DNA methylation regulate lncRNA expression to facilitate target gene transcription to reprogram sex differentiation in *M.albus.*In the future, function of the lncRNA will be further studied and the gene editing technology will be applied to cultivate the female with high fecundity to improve the yield of fish fry.

### Supplementary Information

**Additional file 1: Fig. S1.** Scheme of HE section from developing gonad. OV: ovary; OVT: ovotestis; TE: testis

**Additional file 2: Fig. S2.** Verification of the candidate genes.

**Additional file 3: Fig. S3.** Characterization of candidate transcript in *Monopterus* *albus.* A. the full-length RNA sequence of MSTRG.38036; B. Schematic view of the chromosomal location of MSTRG.38036; C. the full-length RNA sequence of MSTRG.12998; D. Schematic view of the chromosomal location of MSTRG.12998.

**Additional file 4: Fig. S4.** In situ hybridization using sense probe.

**Additional file 5: Fig S5.** Expression of Ptk2β gene after ZD6474 treatment in vivo at different time points. *indicate significantly difference between the two group.

**Additional file 6: Table S1.** Primers used in this study. **Table S3.** Methylation level of each site in the promoter region of lncRNA MSTRG.12998. **Table S4.** methylation level of each site in the promoter region of lncRNA MSTRG.38036. **Table S5.** Methylation level of each site in the promoter region of lncRNA MSTRG.12998 between 17α-methyltestosterone treatment group and control. **Table S6**. Methylation level of each site in the promoter region of lncRNA MSTRG.38036 between 17α-methyltestosterone treatment group and control.

**Additional file 7: Table S2.** List of lncRNAs and its target genes located the same chromosome with co-expression profile

## Data Availability

All data relevant to the study are included in the article or uploaded as supplementary information. The raw sequence data reported in this paper have been deposited in the National Genomics Data Center (National Genomics Data Center and Partners, 2020), Beijing Institute of Genomics (China National Center for Bioinformation), Chinese Academy of Sciences, under the accession number CRA007125 for RNA-seq data.

## Abbreviations

DEGs: Differently expression gene
DELncRNA: Differently expression lncRNA
OVT/OV: Ovotestis/ovary group
TE/ OV: Testis/ovary group
EGFR: Epidermal growth factor receptor
MT: 17α-Methyltestosterone
*Ptk2β*: Protein-tyrosine kinase 2-beta
*Psm**β8*: Proteasome subunit beta type-8

## References

[1] Liu C (1944). Rudimentary hermaphroditism in the symbranchoid eel Monopterus Javanensis. Sinensia.

[2] Qu XC, Jiang JY, Cheng C, Feng L, Liu QG (2015). Cloning and transcriptional expression of a novel gene during sex inversion of the rice field eel (*Monopterus*
*albus*). Springerplus.

[3] Chen H, Liu H, Li R, Lin X, Luo D (2021). Blood cell identification and hematological analysis during natural sex reversal in rice field eel (*Monopterus*
*albus*). Aquaculture.

[4] Tao YX, Lin HR, Van Der Kraak G, Peter RE (1993). Hormonal induction of precocious sex reversal in the ricefield eel, *Monopterus*
*albus*. Aquaculture..

[5] Xiao Q, Sun Y, Liang X, Zhang L, Onxayvieng K, Li Z (2019). Visualizing primordial germ cell migration in embryos of rice field eel (*Monopterus*
*albus*) using fluorescent protein tagged 3' untranslated regions of nanos3, dead end and vasa. Comp Biochem Physiol B Biochem Mol Biol.

[6] Ding W, Cao L, Cao Z, Bing X (2020). Transcriptome analysis of blood for the discovery of sex-related genes in ricefield eel Monopterus albus. Fish Physiol Biochem.

[7] Cai JF, Yang W, Chen D, Zhang YZ, He Z, Zhang WM, et al. Transcriptomic analysis of the differentiating ovary of the protogynous ricefield eel *Monopterus**albus*. BMC Genomics. 2017;18573. 10.1186/s12864-017-3953-6 PMC5541746 28768496

[8] Puvvula PK (2019). LncRNAs regulatory networks in cellular Senescence. Int J Mol Sci.

[9] Marques AC, Ponting CP (2009). Catalogues of mammalian long noncoding RNAs: modest conservation and incompleteness. Genome Biol.

[10] Pang KC, Frith MC, Mattick JS (2006). Rapid evolution of noncoding RNAs: lack of conservation does not mean lack of function. Trends Genet.

[11] Ponting CP, Oliver PL, Reik W (2009). Evolution and functions of long noncoding RNAs. Cell.

[12] Quinn JJ, Chang HY (2016). Unique features of long non-coding RNA biogenesis and function. Nat Rev Genet.

[13] Abdelmohsen K, Panda AC, Kang MJ, Guo R, Kim J, Grammatikakis I (2014). 7SL RNA represses p53 translation by competing with HuR. Nucleic Acids Res.

[14] Tsai MC, Manor O, Wan Y, Mosammaparast N, Wang JK, Lan F (2010). Long noncoding RNA as modular scaffold of histone modification complexes. Science.

[15] LaPak KM, Burd CE (2014). The molecular balancing act of p16(INK4a) in cancer and aging. Mol Cancer Res.

[16] Cai J, Li L, Song L, Xie L, Luo F, Sun S (2019). Effects of long term antiprogestine mifepristone (RU486) exposure on sexually dimorphic lncRNA expression and gonadal masculinization in Nile tilapia (*Oreochromis*
*niloticus*). Aquat Toxicol.

[17] Feng B, Li S, Wang Q, Tang L, Huang F, Zhang Z (2021). lncRNA DMRT2-AS acts as a transcriptional regulator of dmrt2 involving in sex differentiation in the Chinese tongue sole (*Cynoglossus*
*semilaevis*). Comp Biochem Physiol B Biochem Mol Biol.

[18] Leiva F, Rojas-Herrera M, Reyes D, Bravo S, Garcia KK, Moya J, et al. Identification and characterization of miRNAs and lncRNAs of coho salmon (*Oncorhynchus**kisutch*) in normal immune organs. Genomics. 2020;112,45–54. 10.1016/j.ygeno.2019.07.015 31376527

[19] Li L, Jia X, Liu Y, He Y, Pang Y, Shen Y (2021). lncRNA-SUMO3 and lncRNA-HDMO13 modulate the inflammatory response by binding miR-21 and miR-142a-3p in grass carp. Dev Comp Immunol.

[20] He Z, Ye L, Yang D, Ma Z, Deng F, He Z (2022). Identification, characterization and functional analysis of gonadal long noncoding RNAs in a protogynous hermaphroditic teleost fish, the ricefield eel (*Monopterus*
*albus*). BMC Genomics.

[21] Hu QM, Tian HF, Xiao HB (2019). Effects of temperature and sex steroids onsexratio, growth, andgrowth-related gene expression in the Chinese giant salamande*r*
*Andrias*
*davidian*us. Aquat Biol.

[22] Mao X, Cai T, Olyarchuk JG, Wei L (2005). Automated genome annotation and pathway identification using the KEGG orthology (KO) as a controlled vocabulary. Bioinformatics..

[23] Tian HF, Hu QM, Li Z. A high-quality de novo genome assembly of one swamp eel (*Monopterus**albus*) strain with PacBio and Hi-C sequencing data. G3 (Bethesda). 2021;11:jkaa032. 10.1093/g3journal/jkaa032 PMC8022708 33561235

[24] Hu Q, Xiao H, Tian H, Meng Y (2016). Characterization and expression of cyp19a gene in the Chinese giant salamander *Andrias*
*davidianus*. Comp Biochem Physiol B Biochem Mol Biol.

[25] Zhou Y, Zhang X, Xiong S, Zeng X, Zhang X (2021). Predicted gene 31453 (Gm31453) and the gene encoding carboxypeptidase A5 (Cpa5) are not essential for spermatogenesis and male fertility in the mouse. Reprod Fertil Dev.

[26] Hu Q, Lian Z, Xia X, Tian H, Li Z (2022). Integrated chromatin accessibility and DNA methylation analysis to reveal the critical epigenetic modification and regulatory mechanism in gonadal differentiation of the sequentially hermaphroditic fish Monopterus albus. Biol Sex Differ.

[27] Ge C, Ye J, Weber C, Sun W, Zhang H, Zhou Y (2018). The histone demethylase KDM6B regulates temperature-dependent sex determination in a turtle species. Science.

[28] Chen S, Zhang G, Shao C, Huang Q, Liu G, Zhang P (2014). Whole-genome sequence of a flatfish provides insights into ZW sex chromosome evolution and adaptation to a benthic lifestyle. Nat Genet.

[29] Bassett AR, Akhtar A, Barlow DP, Bird AP, Brockdorff N, Duboule D (2014). Considerations when investigating lncRNA function in vivo. Elife.

[30] Sang LJ, Ju HQ, Liu GP, Tian T, Ma GL, Lu YX (2018). LncRNA CamK-A regulates Ca2+-Signaling-Mediated tumor microenvironment remodeling. Mol Cell.

[31] Wang J, Zhang Y, Li Q, Zhao J, Yi D, Ding J (2019). Influenza virus exploits an interferon-Independent lncRNA to preserve viral RNA synthesis through stabilizing viral RNA polymerase PB1. Cell Rep.

[32] Cajigas I, Chakraborty A, Swyter KR, Luo H, Bastidas M, Nigro M (2018). The Evf2 Ultraconserved enhancer lncRNA functionally and spatially organizes megabase distant genes in the developing forebrain. Mol Cell.

[33] Zou X, Wang J, Qu H, Lv XH, Shu DM, Wang Y (2020). Comprehensive analysis of miRNAs, lncRNAs, and mRNAs reveals potential players of sexually dimorphic and left-right asymmetry in chicken gonad during gonadal differentiation. Poult Sci.

[34] Ma X, Cen S, Wang L, Zhang C, Wu L, Tian X (2020). Genome-wide identification and comparison of differentially expressed profiles of miRNAs and lncRNAs with associated ceRNA networks in the gonads of Chinese soft-shelled turtle. Pelodiscus sinensis BMC Genomics.

[35] Nakagawa S, Shimada M, Yanaka K, Mito M, Arai T, Takahashi E (2014). The lncRNA Neat1 is required for corpus luteum formation and the establishment of pregnancy in a subpopulation of mice. Development.

[36] He Z, Ma Z, Yang D, Chen Q, He Z, Hu J (2022). Circular RNA expression profiles and CircSnd1-miR-135b/c-foxl2 axis analysis in gonadal differentiation of protogynous hermaphroditic ricefield eel *Monopterus*
*albus*. BMC Genomics..

[37] Clemson CM, McNeil JA, Willard HF, Lawrence JB (1996). XIST RNA paints the inactive X chromosome at interphase: evidence for a novel RNA involved in nuclear/chromosome structure. J Cell Biol..

[38] Simon MD, Pinter SF, Fang R, Sarma K, Rutenberg-Schoenberg M, Bowman SK (2013). High-resolution Xist binding maps reveal two-step spreading during X-chromosome inactivation. Nature.

[39] Engreitz JM, Pandya-Jones A, McDonel P, Shishkin A, Sirokman K, Surka C (2013). The Xist lncRNA exploits three-dimensional genome architecture to spread across the X chromosome. Science.

[40] Ganesh S, Horvat F, Drutovic D, Efenberkova M, Pinkas D, Jindrova A (2020). The most abundant maternal lncRNA Sirena1 acts post-transcriptionally and impacts mitochondrial distribution. Nucleic Acids Res.

[41] Wichman L, Somasundaram S, Breindel C, Valerio DM, McCarrey JR, Hodges CA (2017). Dynamic expression of long noncoding RNAs reveals their potential roles in spermatogenesis and fertility. Biol Reprod.

[42] Zhu Y, Lin Y, He Y, Wang H, Chen S, Li Z (2020). Deletion of lncRNA5512 has no effect on spermatogenesis and reproduction in mice. Reprod Fertil Dev..

[43] Dai YB, Lin Y, Song N, Sun F (2019). LncRNA4667 is dispensable for spermatogenesis and fertility in mice. Reprod Dev Med.

[44] Li C, Shen C, Shang X, Tang L, Xiong W, Ge H (2020). Two novel testis-specific long noncoding RNAs produced by 1700121C10Rik are dispensable for male fertility in mice. J Reprod Dev.

[45] Otsuka K, Matsubara S, Shiraishi A, Takei N, Satoh Y, Terao M, et al. A testis-specific long noncoding RNA, start, is a regulator of steroidogenesis in mouse leydig cells. Front Endocrinol (Lausanne). 2021;12. 10.3389/fendo.2021.665874 PMC8061315 33897623

[46] Lewandowski JP, Dumbović G, Watson AR, Hwang T, Jacobs-Palmer E, Chang N (2020). The Tug1 lncRNA locus is essential for male fertility. Genome Biol.

[47] Tang L, Huang F, You W, Poetsch A, Nóbrega RH, Power DM (2022). ceRNA crosstalk mediated by ncRNAs is a novel regulatory mechanism in fish sex determination and differentiation. Genome Res.

[48] Chen S, Zhang G, Shao C, Huang Q, Liu G, Zhang P (2014). Whole-genome sequence of a flatfish provides insights into ZW sex chromosome evolution and adaptation to a benthic lifestyle. Nat Genet.

[49] Smith CA, Roeszler KN, Ohnesorg T, Cummins DM, Farlie PG, Doran TJ (2009). The avian Z-linked gene DMRT1 is required for male sex determination in the chicken. Nature.

[50] Jiang YJ, Luo HR, Hou MX, Chen J, Tao BB, Zhu ZY (2022). Aromatase inhibitor induces sex reversal in the protogynous hermaphroditic rice field eel (*monopterus*
*albus*). Aquaculture.

[51] Navarro-Martín L, Viñas J, Ribas L, Díaz N, Gutiérrez A, Di Croce L (2011). DNA methylation of the gonadal aromatase (cyp19a) promoter is involved in temperature-dependent sex ratio shifts in the European sea bass. PLoS Genet.

[52] Heard E, Rougeulle C, Arnaud D, Avner P, Allis CD, Spector DL (2001). Methylation of histone H3 at Lys-9 is an early mark on the X chromosome during X inactivation. Cell.

[53] Drevs J, Konerding MA, Wolloscheck T, Wedge SR, Ryan AJ, Ogilvie DJ (2004). The VEGF receptor tyrosine kinase inhibitor, ZD6474, inhibits angiogenesis and affects microvascular architecture within an orthotopically implanted renal cell carcinoma. Angiogenesis.

[54] Sandström M, Johansson M, Bergström P, Bergenheim AT, Henriksson R (2008). Effects of the VEGFR inhibitor ZD6474 in combination with radiotherapy and temozolomide in an orthotopic glioma model. J Neurooncol.

