# Supplementary figures and images for: Regulatory mechanism of LncRNAs in gonadal differentiation of hermaphroditic fish, Monopterus albus
Source: Biol Sex Differ. 2023 Oct 25;14:74. doi: 10.1186/s13293-023-00559-y (PMC10598917; doi:10.1186/s13293-023-00559-y)

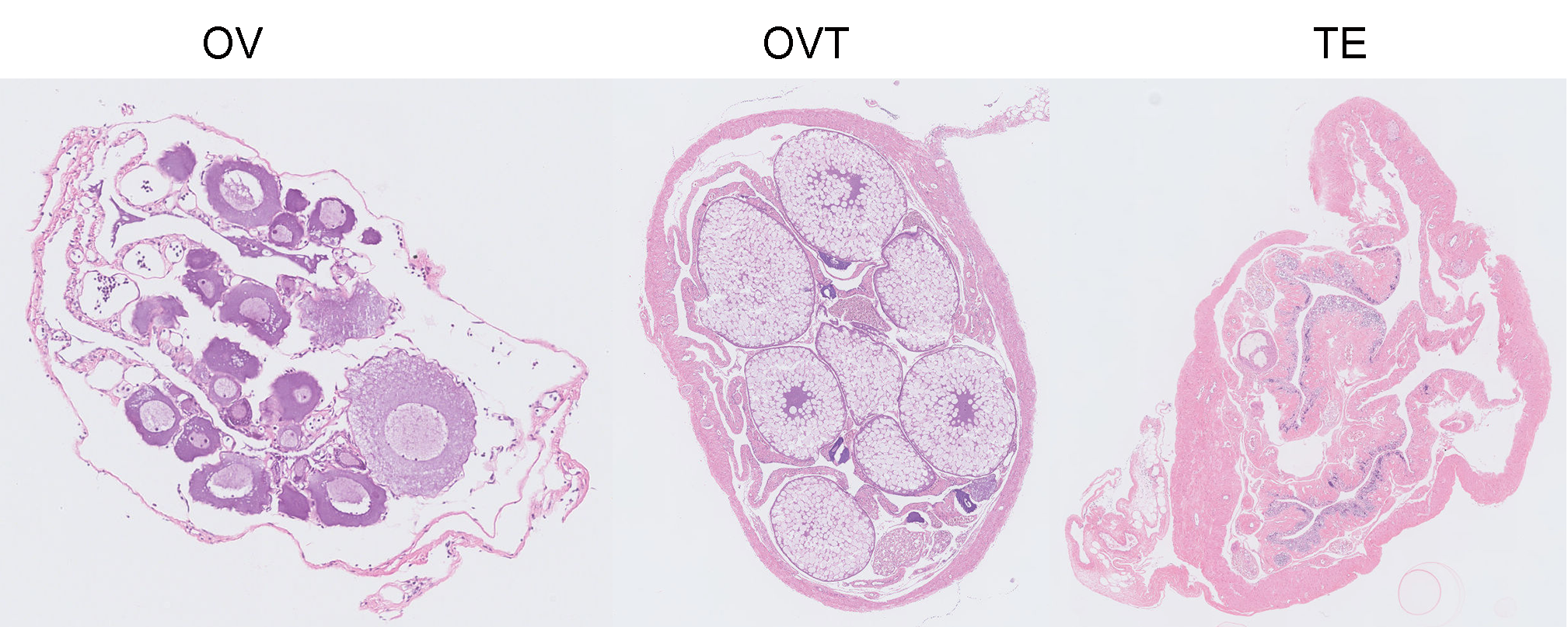

Supplement: Supplementary file 1 — Additional file 1: Fig. S1. Scheme of HE section from developing gonad. OV: ovary; OVT: ovotestis; TE: testis [file 13293_2023_559_MOESM1_ESM.tif]

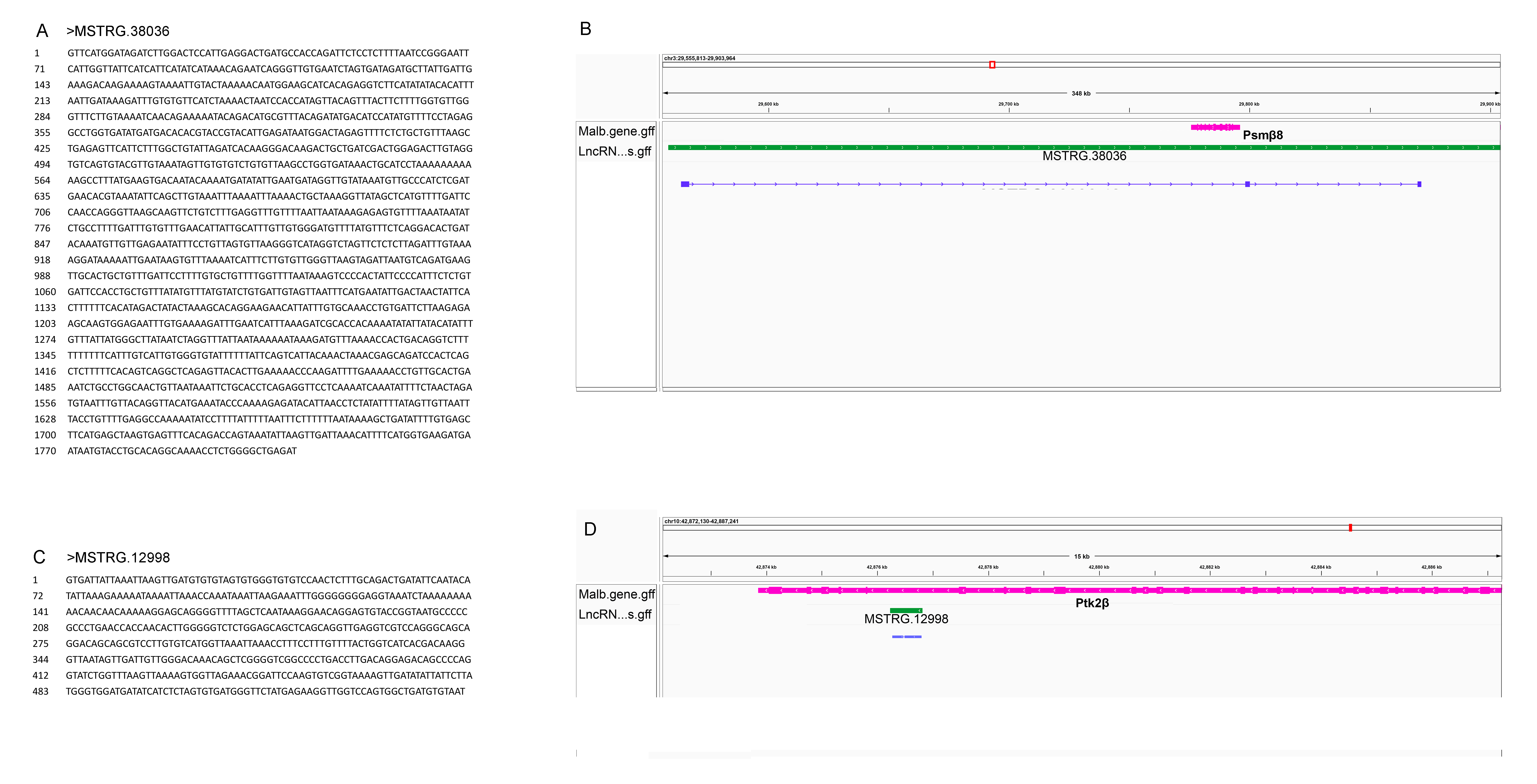

Supplement: Supplementary file 3 — Additional file 3: Fig. S3. Characterization of candidate transcript in Monopterus albus. A. the full-length RNA sequence of MSTRG.38036; B. Schematic view of the chromosomal location of MSTRG.38036; C. the full-length RNA sequence of MSTRG.12998; D. Schematic view of the chromosomal location of MSTRG.12998. [file 13293_2023_559_MOESM3_ESM.tif]

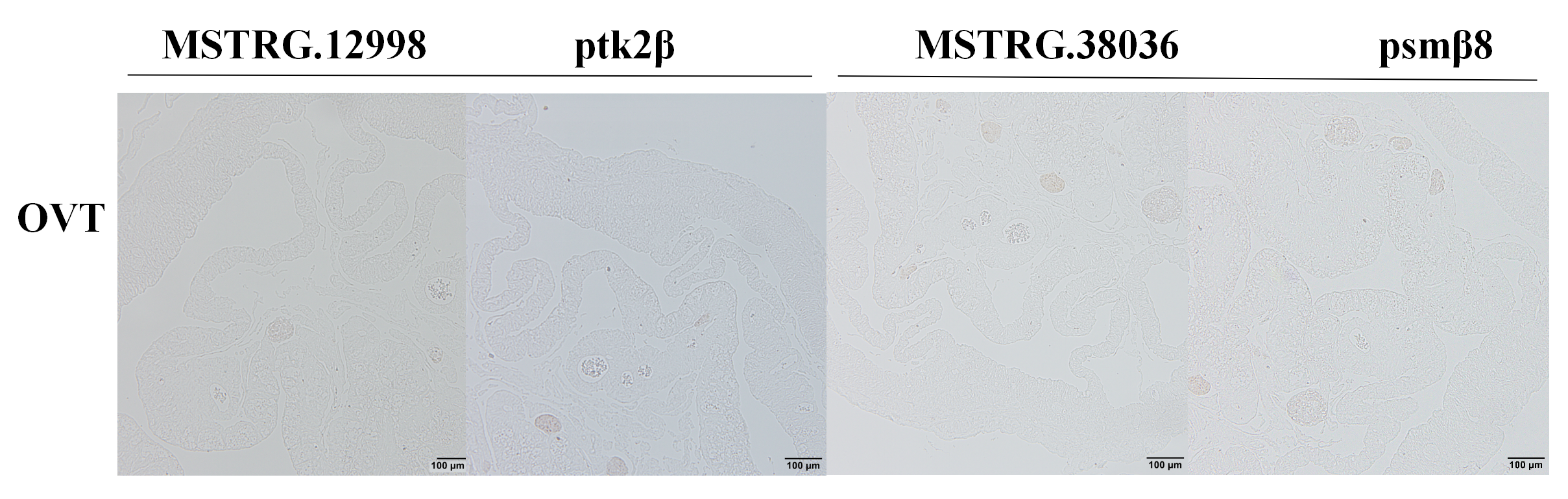

Supplement: Supplementary file 4 — Additional file 4: Fig. S4. In situ hybridization using sense probe. [file 13293_2023_559_MOESM4_ESM.tif]

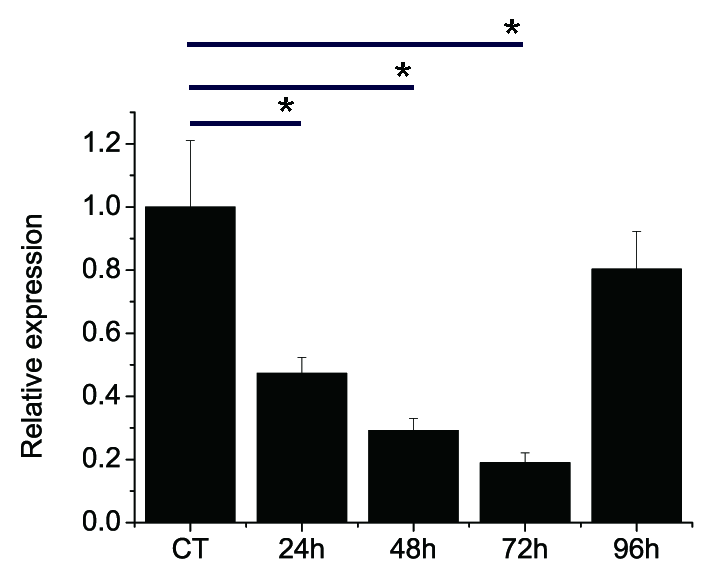

Supplement: Supplementary file 5 — Additional file 5: Fig S5. Expression of Ptk2β gene after ZD6474 treatment in vivo at different time points. *indicate significantly difference between the two group. [file 13293_2023_559_MOESM5_ESM.tif]
