# Supplementary material for: Regulatory mechanism of LncRNAs in gonadal differentiation of hermaphroditic fish, Monopterus albus
Source: Biol Sex Differ. 2023 Oct 25;14:74. doi: 10.1186/s13293-023-00559-y (PMC10598917; doi:10.1186/s13293-023-00559-y)
Supplement: Supplementary file 6 — Additional file 6: Table S1. Primers used in this study. Table S3. Methylation level of each site in the promoter region of lncRNA MSTRG.12998. Table S4. methylation level of each site in the promoter region of lncRNA MSTRG.38036. Table S5. Methylation level of each site in the promoter region of lncRNA MSTRG.12998 between 17α-methyltestosterone treatment group and control. Table S6. Methylation level of each site in the promoter region of lncRNA MSTRG.38036 between 17α-methyltestosterone treatment group and control. [file 13293_2023_559_MOESM6_ESM.doc]

**Regulatory mechanism of LncRNAs in gonadal differentiation of hermaphroditic fish, *Monopterus albus.***

Qiaomu Hu*, Xueping Xia, Zitong Lian,Haifeng Tian, Zhong Li*

Yangtze River Fisheries Research Institute, Chinese Academy of Fishery Sciences, Wuhan 430223, China

*Corresponding authors.

E-mail addresses: [hqmu0806@163.com (QM](mailto:hqmu0806@163.com(QM).H)

lizhong@yfi.ac.cn (Z. Li),

Table S1 Primers used in this study.

| Primers | Primer sequences(5’-3’) | Utilizations |
| --- | --- | --- |
| MSTRG.38036S | TTTGTTGTGGGATGTTTTATGTTTC | qRT-PCR |
| MSTRG.38036A | GAAATGGGGAATAGTGGGGACT | qRT-PCR |
| MSTRG.12998S | CAGGGCAGCAGGACAGCA | qRT-PCR |
| MSTRG.12998A | TGGAATCCGTTTCTAACCACTTT | qRT-PCR |
| MSTRG.31117S | AATGTCGCACAGGTTTCCAGTT | qRT-PCR |
| MSTRG.31117A | AGCCTGATTGGAGATTCCTTACAC | qRT-PCR |
| MSTRG.79384S | GGTTCACAGAGTAAGTTGCCGTAG | qRT-PCR |
| MSTRG.79384A | AAGCAGCCCAGACGCACC | qRT-PCR |
| MSTRG.21895S | TGCCAGCCCATGTATAAACAATA | qRT-PCR |
| MSTRG.21895A | ATTTGCACCAGATTAAAGACCCA | qRT-PCR |
| MSTRG.51212S | AACGCTAAATCAGTGGGAGACC | qRT-PCR |
| MSTRG.51212A | CAACGGAGTAGACTGAACATAAGCA | qRT-PCR |
| MSTRG.40786S | CAGAGGGCAGGAAGAGGGAC | qRT-PCR |
| MSTRG.40786A | TTCCTTATCGACAGACGTTTGGT | qRT-PCR |
| MSTRG.67660S | GTTGAGGACCTGATTGTGCTACC | qRT-PCR |
| MSTRG.67660A | CCAATCAATAAACCTCCAGCAGT | qRT-PCR |
| MSTRG.44101S | CTCCAAAGAAAATGAAGTGCTGTC | qRT-PCR |
| MSTRG.44101A | GCAGCCTTTGCTACCTGTGAA | qRT-PCR |
| MSTRG.24970S | CCTACAGACGAAGCCACTGAACT | qRT-PCR |
| MSTRG.24970 A | TCCCTGCTTGCGTCTTTAGTG | qRT-PCR |
| MSTRG.790S | CCTAAGGGCACTTTGCTAGTTTCA | qRT-PCR |
| MSTRG.790A | CGTATCATTAGGTTTTGGGCTGT | qRT-PCR |
| MSTRG.46549S | GGACTAAATAAAATGGTCGTGGAA | qRT-PCR |
| MSTRG.46549A | TTGCTTTGTTTGATGCTTGACC | qRT-PCR |
| MSTRG.22202S | TCTGCCCAGTGCCGAACA | qRT-PCR |
| MSTRG.22202A | TCTGGAGGGGACTGGCCT | qRT-PCR |
| MSTRG.34402S | TTGGTGCTGTGACCTTCGG | qRT-PCR |
| MSTRG.34402A | AGCCTGGTGATGTCATAGCATTC | qRT-PCR |
| MSTRG.44131S | AGCAAATGCGGTGGGTGA | qRT-PCR |
| MSTRG.44131A | AGAAGCTGAAAGAAGGCGGTAG | qRT-PCR |
| Psmβ8S | AGCCTCCGCTGGAAACTACC | qRT-PCR |
| Psmβ8A | ATCATGTTTGACAGCAGCTTGG | qRT-PCR |
| Ptk2βS | TCCTGGGCGAGGGGTTC | qRT-PCR |
| Ptk2βA | TGACTCCGATTAGACGCACGA | qRT-PCR |
| Mal0028450S | TTCTGCCACTCTGCCCATTAC | qRT-PCR |
| Mal0028450A | ACTGGCTGCTGTGGCTGTAATA | qRT-PCR |
| Mal0036330S | GAGATTTCGCTGAGAATTGGTACA | qRT-PCR |
| Mal0036330A | CAGATAGCGGGCTTCTTTACG | qRT-PCR |
| Mal0067860S | CTGCCTCCTCATCTCCTACACC | qRT-PCR |
| Mal0067860A | AGATGGAGAAGCAGATGATGAAGAC | qRT-PCR |
| Mal0045660S | ACTGCGGTGGCTTCCTTCT | qRT-PCR |
| Mal0045660A | GTTCCGTTGAAGTCTTTATGTGGA | qRT-PCR |
| Mal0205680S | GGCATCACAGCAGCCGAC | qRT-PCR |
| Mal0205680A | GTCCACCATCTGATTGTTCGTTT | qRT-PCR |
| Mal0187090S | TGCTGAAGGGCACCATAAAGA | qRT-PCR |
| Mal0187090A | TGTTGGGTTTCAGTGACTTTTCC | qRT-PCR |
| newGene_24860S | TGGTTACAGCACTGCCAAATAAG | qRT-PCR |
| newGene_24860A | CCTTAGCTTTCACAACGCCAT | qRT-PCR |
| newGene_11061S | TTTGAGCAGGAAATGTGGTGG | qRT-PCR |
| newGene_11061A | ACATAAGGTGGGTTTACTGTGGC | qRT-PCR |
| newGene_48717S | ATGGAAGGATAAATGCTGGGTC | qRT-PCR |
| newGene_48717A | GTTTCGTTCGGCTCACTTACACT | qRT-PCR |
| Mal0109580S | CACAACTCTGCGGTCCCTCTA | qRT-PCR |
| Mal0109580A | CAGCGATGTTGAGGAGGACC | qRT-PCR |
| Mal0129700S | GATTCTGGGACGGAGGTGG | qRT-PCR |
| Mal0129700A | TCCTCTTCTTCTTGCCGTAGTTG | qRT-PCR |
| newGene_17932S | ATGGGAGGTGTTTGGGAGC | qRT-PCR |
| newGene_17932A | TTGGAGTAAGTGGCTCTAAACCTT | qRT-PCR |
| Mal0133410S | GAGGTGAAACACTTCTGCCCTAA | qRT-PCR |
| Mal0133410A | GTCCCGTCCATCCTCTGGTT | qRT-PCR |
| Cyp19a1S | CGTCGAGCCCTGACCGAT | qRT-PCR |
| Cyp19a1A | TGAACCGAATGGCTGGAAGT | qRT-PCR |
| EGFRS | ACAGAACAGACCGTCTTGGTAGTTT | qRT-PCR |
| EGFRA | GGACTGTCACCCCGAATGC | qRT-PCR |
| STAT3-S | GCTATAAGATTATGGATGCAACCAA | qRT-PCR |
| STAT3-A | AGGTATGGCTGAATAGTGCTCGTA | qRT-PCR |
| Dmrt1aS | CCCAGGATGCCCAAGTGC | qRT-PCR |
| Dmrt1aA | TCTCGGCTATCAGTTTACATTTGG | qRT-PCR |
| EF-1a-F2 | CGCTGCTGTTTCCTTCGTCC | qRT-PCR |
| EF-1a-R2 | TTGCGTTCAATCTTCCATCCC | qRT-PCR |
| LncR38036 pro/methS | TGTGGTTTGATTGGTAGATAGATG | DNA methylation |
| LncR38036pro/methA | AATTCAATTATTCCATAACCTCAAA | DNA methylation |
| LncR12998Pro/methS | TTGTAGGCGGTGGGGGTT | DNA methylation |
| LncR12998Pro/methA2 | CTACCGCTAACACTCCTCCTT | DNA methylation |
| MSTRG.38036S | TTTGTTGTGGGATGTTTTATGTTTC | In situ hybridization |
| MSTRG.38036A-T7 | GATCACTAATACGACTCACTATAGAAGTGTAACTCTGAGCCTGACTGTG | In situ hybridization |
| MSTRG.38036S-T7 | GATCACTAATACGACTCACTATAGTTTGTTGTGGGATGTTTTATGTTTC | In situ hybridization |
| MSTRG.38036A | AAGTGTAACTCTGAGCCTGACTGTG | In situ hybridization |
| Psmβ8S | GCACCTCGTAGACCGAACCA | In situ hybridization |
| Psmβ8A-T7 | GATCACTAATACGACTCACTATAGCCCAGTTCATACGCCTCCTC | In situ hybridization |
| Psmβ8S-T7 | GATCACTAATACGACTCACTATAGGCACCTCGTAGACCGAACCA | In situ hybridization |
| Psmβ8A | CCCAGTTCATACGCCTCCTC | In situ hybridization |
| Ptk2βS | TCACGCAACACGATATTTGTCC | In situ hybridization |
| Ptk2βA-T7 | GATCACTAATACGACTCACTATAGTCCCCTAGATCCTTGTTTAGCAGT | In situ hybridization |
| Ptk2βS-T7 | GATCACTAATACGACTCACTATAGTCACGCAACACGATATTTGTCC | In situ hybridization |
| Ptk2βA | TCCCCTAGATCCTTGTTTAGCAGT | In situ hybridization |
| MSTRG.12998S | CCAACTCTTTGCAGACTGATATTCA | In situ hybridization |
| MSTRG.12998A-T7 | GATCACTAATACGACTCACTATAGCAGCCACTGGACCAACCTTC | In situ hybridization |
| MSTRG.12998S-T7 | GATCACTAATACGACTCACTATAGCCAACTCTTTGCAGACTGATATTCA | In situ hybridization |
| MSTRG.12998A | CAGCCACTGGACCAACCTTC | In situ hybridization |
| PGL3-Cyp19a1pro1S | GGCGGTACCTGTCACTCATTCACTGTGGTT | Plasmid construction |
| PGL3-Cyp19a1pro1A | GGCAAGCTTTAAAAAAAAAAAAAATATATATATATATATAT | Plasmid construction |
| PGL3-Cyp19a1pro2S | GGCGGTACCGAGTAACAGATCTTTCAAGTTATAG | Plasmid construction |
| PGL3-Cyp19a1pro2A | GGCAAGCTTTAAAAAAAAAAAAAATATATATATATATATA | Plasmid construction |
| PGL3-Cyp19a1pro3S | GGCGGTACCGCAAACTGCATGGGGTCTGT | Plasmid construction |
| PGL3-Cyp19a1pro3A | GGCAAGCTTTAAAAAAAAAAAAATATATATATATATATATAGAT | Plasmid construction |
| pcDNA3.1-STAT3S | GGCGCTAGCATGGCCCAGTGGAACCAGTTA | Plasmid construction |
| pcDNA3.1-STAT3A | GGCCTCGAGTCACATAGGTGAAGCTACATCCATG | Plasmid construction |
| Cyp19a1pro3/mut1S | GGCTGAAAGAACATCGAGACATGATGTGGAAGG | Site mutation |
| Cyp19a1pro3/mut1A | CCTTCCACATCATGTCTCGATGTTCTTTCAGCC | Site mutation |
| Cyp19a1pro3/mut2S | ATCTGAGATCTGTCTGCTGCAGAGTGCAGTGCTG | Site mutation |
| Cyp19a1pro3/mut2A | CAGCACTGCACTCTGCAGCAGACAGATCTCAGAT | Site mutation |

Table S3 methylation level of each site in the promoter region of lncRNA MSTRG.12998

|  | 1 | 2 | 3 | 4 | 5 | 6 | 7 | 8 |
| --- | --- | --- | --- | --- | --- | --- | --- | --- |
|  | -8 | -25 | -48 | -75 | -290 | -592 | -620 | -640 |
| OV | 100 | 93.3 | 100 | 100 | 100 | 80 | 80 | 100 |
| OVT | 100 | 87.5 | 87.5 | 81.2 | 100 | 81.2 | 87.5 | 100 |
| TE | 100 | 76.9 | 76.9 | 84.6 | 76.9 | 84.6 | 76.9 | 92.3 |

Table S4 methylation level of each site in the promoter region of lncRNA MSTRG.38036

|  | 1 | 2 | 3 | 4 | 5 | 6 | 7 | 8 | 9 |
| --- | --- | --- | --- | --- | --- | --- | --- | --- | --- |
|  | -103 | -121 | -142 | -201 | -272 | -284 | -344 | -387 | -410 |
| OV | 86.7 | 91.7 | 86.7 | 93.3 | 100 | 100 | 86.7 | 93.3 | 73.3 |
| OVT | 93.3 | 78.6 | 69.2 | 80 | 93.3 | 80 | 92.3 | 92.9 | 86.7 |
| TE | 84.6 | 90 | 92.3 | 100 | 100 | 84.6 | 100 | 92.3 | 100 |

Table S5 methylation level of each site in the promoter region of lncRNA MSTRG.12998 between 17α-methyltestosterone treatment group and control.

|  | 1 | 2 | 3 | 4 | 5 | 6 | 7 | 8 |
| --- | --- | --- | --- | --- | --- | --- | --- | --- |
|  | -8 | -25 | -48 | -75 | -290 | -592 | -620 | -640 |
| C | 100 | 100 | 81.2 | 87.5 | 100 | 81.2 | 87.5 | 100 |
| MT | 100 | 66.7 | 73.3 | 86.7 | 73.3 | 66.7 | 60 | 100 |

Table S6 methylation level of each site in the promoter region of lncRNA MSTRG.38036 between 17α-methyltestosterone treatment group and control.

|  | 1 | 2 | 3 | 4 | 5 | 6 | 7 | 8 | 9 |
| --- | --- | --- | --- | --- | --- | --- | --- | --- | --- |
|  | -103 | -121 | -142 | -201 | -272 | -284 | -344 | -387 | -410 |
| Control | 92.3 | 61.5 | 84.6 | 84.6 | 92.3 | 92.3 | 92.3 | 100 | 75 |
| MT | 100 | 69.2 | 92.3 | 84.6 | 92.3 | 76.9 | 100 | 100 | 69.2 |
